# Storytelling as narrative health promotion in community psychiatry: a quasi-experimental study

**DOI:** 10.1186/s12888-025-06816-1

**Published:** 2025-04-14

**Authors:** Márk Komóczi, Karolina Kósa

**Affiliations:** Department of Behavioural Sciences, Faculty of Medicine, University of Debrecen, Debrecen, Hungary

**Keywords:** Community psychiatry, Hungary, Intervention, Narrative psychology

## Abstract

**Background:**

Community-based psychiatric rehabilitation (CBPR) helps patients reintegrate into society while enabling them to live autonomously in supportive environments. CBPR uses multi-modal approach to address patients’ needs in health, education, livelihood, empowerment and social functioning. In addition to pharmacotherapy, other interventions such as metacognitive training, lifestyle interventions, psychoeducation, arts therapy may be used to improve functioning and quality of life. Storytelling as a new intervention was implemented in a community-based rehabilitation setting with patients with mental health issues to test its feasibility and potential to improve life satisfaction.

**Methods:**

Stories presenting difficult lives and complicated relationships were narrated and discussed in eight storytelling sessions for members of a civil organization involved in psychiatric rehabilitation in four months. Acceptability was tested by following participation rate and feedback with scales after each session. Demographic as well as mental health data including sense of coherence, distress, self-efficacy, and life satisfaction were investigated by standard scales before the first and after the last session.

**Results:**

Participation ranged from 31 to 49% compared to all persons present at the setting. Participants (mean age: 53.41 ± 12.23 years, 63% females) found the stories highly interesting (mean: 8.93 ± 1.62) and comprehensible (8.67 ± 1.9) on a 1–10 scale though the means of individual sessions somewhat varied. Significant positive correlation was found between the stories being interesting and comprehensible (Spearman’s rho = 0.656) but significant negative correlation was found between story length and comprehension (Spearman’s rho=-0.183). Based on the responses from participants who completed the questionnaires before and after the intervention, life satisfaction significantly increased. Psychological variables such as self-efficacy, sense of coherence, pathological distress showed improving tendency without reaching significance. Pre-intervention data showed significant positive correlation between self-efficacy and sense of coherence (Pearson’s *r* = 0.659). Psychological distress was negatively related to both self-efficacy (Pearson’s *r*=-0.728) and sense of coherence (Pearson’s *r*=-0.825).

**Conclusions:**

Storytelling as a means for promoting health proved to be feasible in a group of rehabilitated patients with mental disorders. Their life satisfaction significantly improved in four months. Carefully selected stories narrated and discussed in group settings may result in the gradual shift of participants’ perspectives leading to improved life satisfaction.

**Supplementary Information:**

The online version contains supplementary material available at 10.1186/s12888-025-06816-1.

## Background

### Concept of community-based psychiatric rehabilitation

The concept of psychiatric rehabilitation arose from several historical developments, one of which was the recognition in the 1950s of the capacity of the mentally retarded for employment [1, 2]. This led to a legislation-based gradual transfer of patients of mental health issues out of hospitals and into community-based services through which they could be helped to rebuild and maintain functioning in as normal a manner as possible with minimal professional help [3]. The experiences of therapeutic communities of patients suffering from similar ailments [4, 5] also contributed to the rise of community-based psychiatric rehabilitation with the double aim of developing the skills of individuals to interact in stressful environments as well as creating environments in which potential stressors are minimized. Considering that persons with lived experience of mental illness have similar aspirations as non-patients, community-based rehabilitation services– in addition to helping patients manage their conditions– also support them in their everyday needs such as finding housing, work opportunities, building and maintaining relationships, and participate in the life of their wider communities as autonomous citizens free from stigma [6, 7].

Community-based mental rehabilitation has been particularly beneficial for persons with schizophrenia who are able to regain autonomous functioning if comprehensive and integrated care is provided [8, 9]. Such services are particularly important for low-income patients so much so that lack of sufficient funding leading to the closure of such services may result in tragic consequences. This was shown by US authors who found a negative association between the number of community mental health centers and suicide deaths between 2014 and 2017 in the country [10]. Community psychiatric rehabilitation services have also been shown to produce improved outcomes when delivered by lay persons [11]. This is an important observation knowing that resources for mental health tend to be insufficient even in high-income countries and are especially inadequate in middle- and low-income countries [12].

Hungary has historic antecedents of community-based psychiatric services. The first experiments with community-based care were carried out in the 1920s when persons with lived experience of mental illness were placed with rural families volunteering to accept them [13]. An inpatient mental hospital was turned into and operated as a therapeutic community between 1952 and 1957 by István Benedek, a humanistic psychiatrist [14]. However, the political ideology of the communist era did not favor progressive initiatives [15] so the establishment of community-based psychiatric services only became possible after the fall of communism. A non-governmental organization (NGO) named Awakenings (Ébredések) [16] established in 1991 opened the way to community-based psychiatric rehabilitation in the country which reflected the international trend to prioritize bottom-up or grassroots approach when establishing community-based services. As of 2023, 32% of all outpatient mental services in Hungary are community-based [17].

### Methods of community-based psychiatric rehabilitation

Community-based rehabilitation (CBR) starts with the strengths and capacities (rather than the deficiencies) of the persons living with mental health problems while using the comprehensive definition of mental health of the World Health Organization which, among others, includes the meaningful contribution to the community as part of mental health [18]. CBR is conducted by multidisciplinary teams with the participation of doctors, psychologists, psychiatrists, social workers, physiotherapists, dietitians etc. building on the active and voluntary contribution of patients. When community services are understaffed or nonexistent, lay health workers with proper supervision and training can effectively provide structured psychological assistance [11, 19].

CBR covers health, education, livelihood, empowerment and social aspects [20]. While pharmacotherapy is essential, CBR should also include disease prevention through screening, assistive devices (e.g., walking sticks, hearing aids) and dietetic consultation because psychiatric medications increase the risk of obesity [21]. Lifestyle interventions help to manage diabetes [22] and weight gain for persons with schizophrenia [23]improving quality of life. All persons with mental health problems, even those living with serious mental illness (SMI) [24] including those with schizophrenia may benefit from art therapy [25], the umbrella term for a wide range of modalities ranging from drama therapy [26] and music therapy [27] to Chinese calligraphy therapy [28] that mobilize patients’ creativity. Dance therapy and movement therapies have positive effects on health and also enhance well-being [29, 30]. Health promoting programs can increase empowerment while reduce stigmatization and social exclusion. Psychoeducation helps patients, families and friends to understand symptoms, treatment and disorder management [31].

Cognitive remediation [32] and metacognitive training (MCT), developed for those living with psychotic disorders, address cognitive distortions in schizophrenia. MCT, developed by Moritz and Woodwards [33], combinescognitive behavior therapy, cognitive remediation and psychoeducation, using real-life scenarios and stories to help the patients recognize and reinterpret cognitive distortions.

Other similar but less structured approaches for improving cognitive skills also employ narratives, that is, fictitious or real-life stories, tales and fables told and discussed in small groups. Narratives can be found everywhere, particularly in myths, tales, novels, history, painting, cinema, and the news. Narratives begin with the history of humanity and influence everyone’s life [34].

A British literary scholar argued in 1968 that the narrative is not an aesthetic invention used by artists but “a primary act of mind transferred to art from life” which affects human cognition since humans dream, remember, believe, plan, hope and love in narratives [35]. This idea was an early precursor to the field of narrative psychology of which Theodore Sarbin was a pioneer. He considered the narrative (or story in its ordinary meaning) as a root metaphor for psychology because an individual’s life story contains the identity, desires, goals, and responsibilities of the person [36]. Jerome Bruner, one of the early representatives of cognitive psychology [37, 38] differentiated between paradigmatic and narrative modes of thought. In the former mode, logical thinking prevails and focuses on what is considered scientifically right or correct. In contrast, the narrative mode aims at sense-making for everyday interpretation [39, 40].

The increasing interest towards narratives resulted in their integration into psychotherapy in various forms such as narrative therapy [41] in which patients create and tell stories, and storytelling in which the therapist shares stories with the patient [42].

The authors of the present paper decided to deliver and test the latter method when they were invited by a non-governmental organization to work with participants of a community-based rehabilitation program organized for persons living with mental disorders. Eight sessions of storytelling were designed and delivered during four months as part of the community-based rehabilitation program with free and voluntary participation for anyone.

Objectives.

1. Assessment of feasibility of storytelling sessions for persons with lived experience of mental illness by evaluating participation and dropout rates.
2. Exploration of participant’s engagement by measuring how interesting and understandable they find the stories.
3. Examination of the impact of storytelling sessions on participants’ mental well-being.

## Methods

### Setting

The Association of Psychotic Patients (APP) [43], a civil organization founded in 1997 operates a community-based psychiatric rehabilitation facility in Nyíregyháza, Szabolcs-Szatmár-Bereg County, Hungary. The Association supports patients with mental health issues and their relatives in the region by providing employment opportunities, educational and health promoting programs as well as social and cultural events. The Association has 94 members who consciously chose the name of the NGO (Association of Psychotic Patients) so as to help de-stigmatize the word and persons associated with it. The president of the Association invited the authors to design and implement a health promoting project for their members funded by a governmental tender.

### Project design

The APP provided freedom for the authors to design the project, specifying only that participation be open for anyone who was interested, duration be no more than two hours per session with a total number of eight sessions delivered on the premises. Members of the Association previously encountered various methods including metacognitive training, psychoeducation, different forms of art and movement therapy and occupational therapy. Considering the diversity of potential participants, their familiarity with a number of rehabilitation methods, and time constraints, the authors planned group sessions of storytelling. Eight two-hour sessions were conducted between November 2023 and February 2024 in the largest meeting room of the APP in Nyíregyháza in Northeastern Hungary. Ethics approval was issued by the Regional Institutional Research Ethics Committee, Clinical Center, University of Debrecen (IKEB/RKEB 6714–2024). All participants gave informed consent before participation.

### Participants

Persons with lived experience of mental disorder, members of the APP present at the facility before the beginning of the session could join each of the sessions. An open group format was chosen so anyone could join any session, regardless of previous presence or absence of precedent sessions. All members of APP were informed about the upcoming sessions by the president during several daily meetings. Members with lived experience of mental disorder were invited to participate anonymously and voluntarily. Before each session, participants signed their consent and presence on a registration sheet that was distributed and collected by the office manager of the APP for documentation purposes (who did not participate in the sessions) without the presence of the authors.

### Intervention

Nondirective sessions of 90–120 min duration, quasi-experimental design were conducted for open groups loosely divided into three parts. After greeting, an oral story was narrated in 10–15 min with no illustration (except in some stories about real persons when pictures of the protagonist were shown). Stories were chosen from the literature and medical literature which described difficult lives, challenging situations, complicated relationships, or relevant decisions made without the protagonist’s consent– in other words, situations most likely also faced by the participants during their lives. One Greek myth, one medieval story, one fantasy tale, and real-life stories of five persons were selected for presentation in the first part of the sessions. Each story had multiple presentations of varying length in printed and online format that were used as sources. Each story was written up into one written narrative with a mean length of 2,600 words which was used as the basis of the free oral (not read) presentation. The number of words of the written narrative was used to characterize the length of the story.

The second part was an open discussion of the story during which participants shared their thoughts on the protagonist’s behavior, thoughts and emotions, and tried to capture the take-home messages of the stories, or they could also ask questions from the authors or other participants. In almost all sessions, at least one participant shared a similar or related story from his/her own life. Anything the participants wished to tell was possible, but no-one was obligated to speak. It took some participants several sessions to share their thoughts. One person suggested a specific story for subsequent sessions. In the third part of the session, participants– without prompting– usually attempted to connect the message of the stories with their life experiences and tried to find the meaning for themselves.

### Methods of data collection

Demographic characteristics including age (date of birth), gender (male/female), marital status, subjective wealth and measures of mental status detailed below were collected by the researchers with anonymous paper-based Hungarian questionnaires.

### General health questionnaire (GHQ-12)

The General Health Questionnaire contains 12 items and estimates psychological distress based on answers rated on a 1–4-point Likert scale [44, 45]. The questionnaire has a total score ranging from 12 to 48, but items can be evaluated as binary variables assigning a score of 1 to each symptom if present and 0 if absent, thereby creating a total score between 0 and 12. We applied the latter method, using the threshold of 4 above which the score reflects pathological distress [46]. The questionnaire has a high reliability, Cronbach’s α was 0.945 before, and 0.946 after the intervention.

### Sense of coherence short version (SOC-13)

This is the abbreviated version of Antonovsky’s 29-item questionnaire of sense of coherence. 13 items are answered on a 1–7 point Likert scale. The lowest total score is 13 and the maximum is 91 [45, 47]. The reliability of the questionnaire was high both before (Cronbach’s α = 0.908) and after (Cronbach’s α = 0.864) the intervention.

### General self-efficacy scale (GSE-10)

The 10-item scale was developed by Schwarzer and Jerusalem was used to assess an optimistic self-belief which predicts coping with daily problems [48]. Answers are given on a 1 to 4 Likert scale yielding a total score ranging from 10 to 40. Higher scores reflect more self-efficacy. The reliability of the questionnaire was high both before (Cronbach’s α = 0.919) and after (Cronbach’s α = 0.898) the intervention.

### Single-item measure of life satisfaction (LS)

Participants rated their current life satisfaction on a scale from 0 (‘not at all’) to 10 (‘totally’) [49].

### Single-item measure of understanding

Participants responded on a scale from 0 (‘not at all’) to 10 (‘totally’) as to how understandable or comprehendible they found the actual story. This question was delivered after each session.

### Single-item measure of interest

Participants responded on a scale from 0 (‘not at all’) to 10 (‘totally’) as to how interesting they found the actual story. This question was delivered after each session.

Since relatives of patients can also be APP members, there was the possibility that they join the sessions. Therefore, one question asked whether participants had a mental disorder (ever had mental disorder), and in case of positive answer, another question related to the time elapsed since the last hospitalization because of the disorder. Table 1 presents the measurements conducted during our project alongside our objectives.

**Table 1:** Collected data by objectives

Measures / questionnaires for assessment	Objectives		
	Objective 1: Assessment of feasibility by monitoring participation	Objective 2: Evaluation of the stories by interest and understanding	Objective 3: Evaluation of mental status a) before vs. gen. pop. b) before-after
Age	yes	yes	yes
Gender	yes	yes	yes
Number of participants at the session	yes		
Actual story being interesting		yes	
Actual story being comprehendible		yes	
Life satisfaction (LS)		yes	yes
Psychological distress (GHQ-12)			yes
Sense of coherence (SOC-13)			yes
Self-efficacy (GSE-10)			yes
Ever had mental disorder			yes
If ever had mental disorder, time since last hospitalization			yes

### Statistical analysis

Statistical analysis was conducted in Jamovi 2.4.11.0 (Sydney, Australia). The two tailed p-value’s level was set at less than 0.05 (*p* < 0.05). The normality of continuous and interval variables was tested by Shapiro-Wilk and Kolmogorov-Smirnov tests. In case of normally distributed values, categories were compared by chi-square test. In case of non-normality, Kruskal-Wallis test was used. Post hoc analysis was carried out with Dwass-Steel-Critchlow-Fligner pairwise comparisons [50].

## Results

### Objective 1: assessment of feasibility by monitoring participation

Throughout the project, a total of 27 individuals participated, including 10 males and 17 females. 8 individuals attended only one session. 4 individuals attended 6 sessions, 5 individuals attended 7 sessions, and 4 responders attended all the sessions. The mean age of the participants was 53.41 ± 12.23, with a median age of 55 years. The youngest participant was 28, while the oldest was 82 years of age.

The first data collection was carried out before the first group session with 19 respondents (mean age 53.95 ± 12.95) of whom 63% were females, and 84% were employed. Of 13 individuals who gave answers for this item, 68% ever had a mental illness, with a mean of 8 years (SD = 8.34, min = 0.5; max = 27) elapsing since their last hospitalization.

**Fig. 1:**
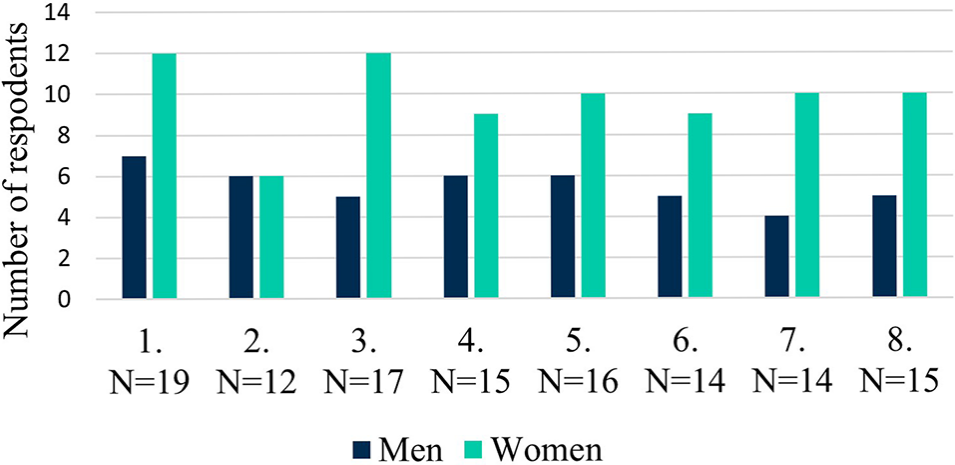
Number of respondents by sessions

The mean of the submitted questionnaires for all 8 sessions was 15.25 (median = 15). The lowest participation was seen in the second session with 12 persons, and the highest number was 20 in the third session (Fig. 1). The participation rate was relatively high ranging from at least of 31% to no more than 49% compared to those present on the premises at the time of the session.

### Objective 2: evaluation of the stories by interest and comprehensibility

When evaluating the stories after the session to answer Objective 2, the response rate was 100% meaning that all participants filled the questionnaire except after session 3 when 17 out of 20 participants (85%) provided feedback. The descriptive statistics of the variables and feedback indicators of the sessions are presented in Appendix 1.

The story of the first session (Greek myth) was rated as the least interesting (mean = 8.16 ± 2.03) and most difficult to comprehend (mean = 7.37 ± 2.31). Participants found one of the real-life stories (the story of a lobotomized patient with mental health issues) the most interesting (mean = 9.44 ± 0.89), and 3 short real-life stories of the last session to be most understandable (mean = 9.71 ± 0.61). Real-life stories (3., 5–8.) had higher mean scores in both variables than fictional stories.

**Fig. 2:**
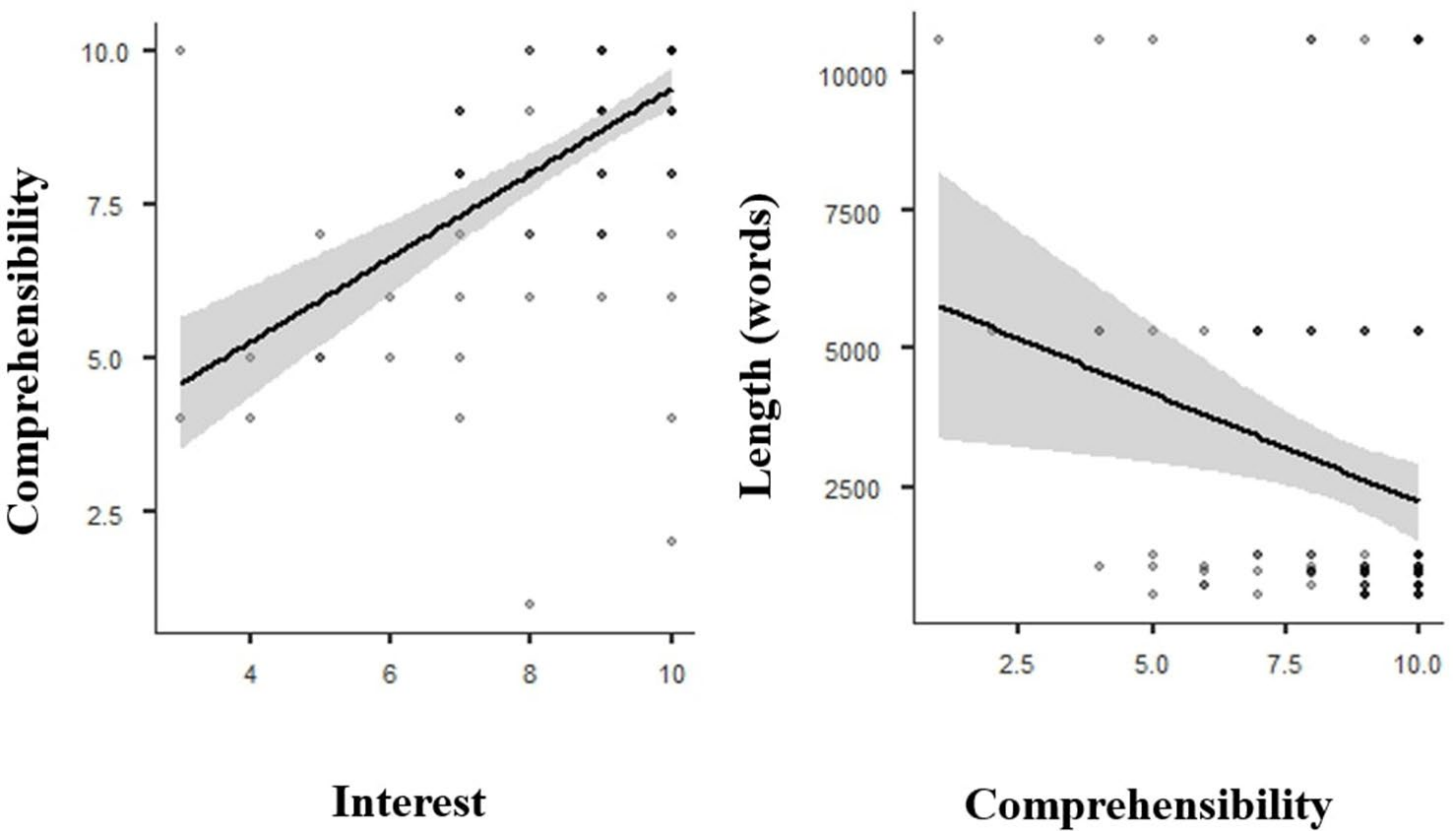
Linear fit models with confidence intervals of comprehensibility, interest and length of the stories

Significant positive correlation was found between the stories being interesting and understandable (Spearman’s rho = 0.656, *p* < 0.001). Significant negative correlation was found between the length of the stories calculated from the number of words of the story in its written form and comprehension (Spearman’s rho=-0,183 *p* = 0.045) (see Fig. 2.) There was no association between length of the story and interest (*p* = 0.095), and there was no correlation between time elapsed from last hospitalization (mean: 91 months) and level of understanding (Spearman’s rho=-0.174 *p* = 0.655).

### Objective 3: evaluation of mental status before and after the project

Variables measuring self-efficacy (GSE-10), sense of coherence (SOC-13), psychological distress (GHQ-12) and life satisfaction (LS) were assessed before the first and after the last session as described in Methods. In order to improve the balance (the comparability) of the sample before and after, questionnaires submitted before and after the project were matched by age, gender, educational level and marital status resulting in a sample of 11 questionnaires before and after whose respondents were likely to be identical. Respondents in this sample participated on average at 7 sessions. Their mean age was 51.18 ± 9.39 years; 64% were females, and 81% acknowledged to have had a mental illness.

All of the descriptive characteristics of the investigated variables were normally distributed with the exception of self-efficacy assessed after the project (Appendix 2). There was a tendency of improvement in all three psychological variables but no significant increase was seen in self-efficacy (*p* = 0.953) or sense of coherence (*p* = 0.567), and the proportion of pathologically distressed did not decrease (*p* = 0.341). However, life satisfaction became significantly increased after the project compared to before by one-tailed paired t-test (*p* = 0.028).

**Fig. 3:**
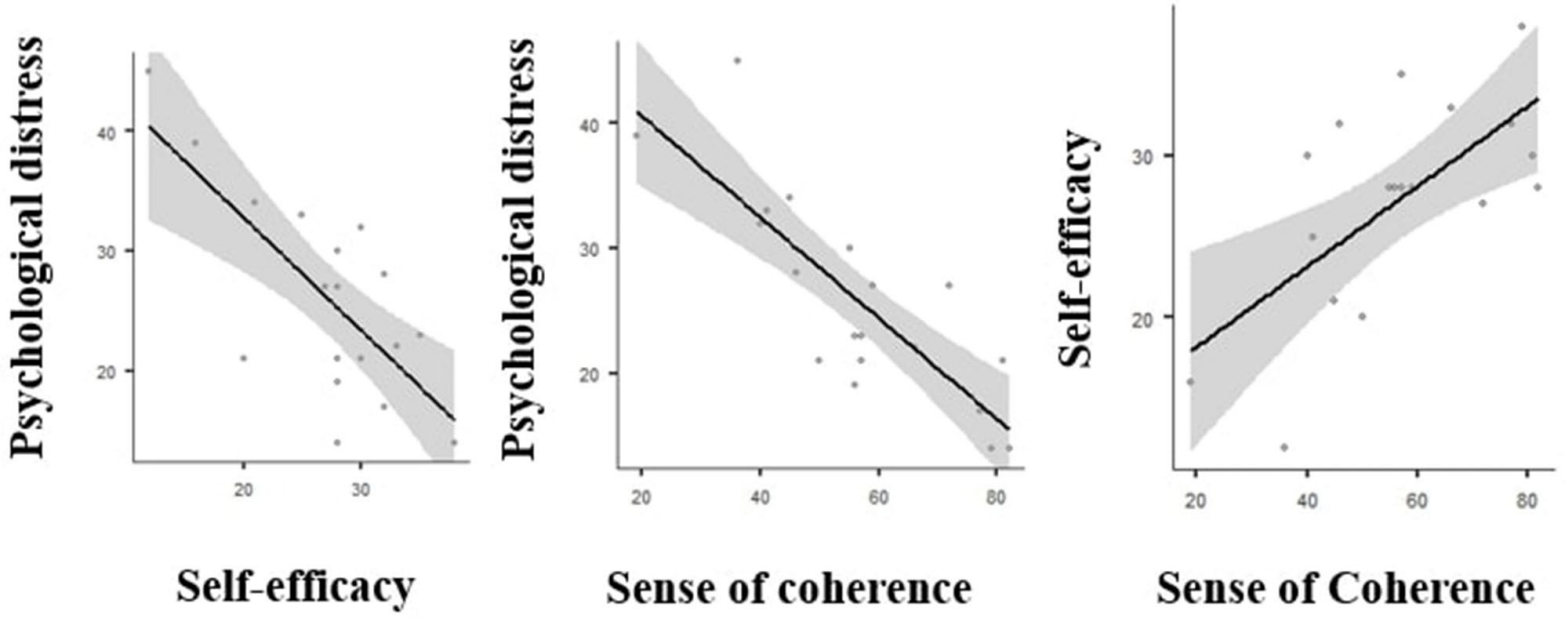
Linear fit models with confidence intervals of the standardized questionnaires

Self-efficacy (GSE-10) and sense of coherence (SOC-13) showed significant positive correlation (Pearson’s *r* = 0.659 *p* = 0.003) Psychological distress (GHQ-12) and self-efficacy (GSE-10) were negatively related (Pearson’s *r*=-0.728 *p* < 0.001), just as distress and sense of coherence (SOC-13) (Pearson’s *r* =-0.825 *p* < 0 0.001) (Fig. 3).

In addition to the before-after comparison, we also compared our pre-intervention respondents (*N* = 19) to a representative sample of the adult Hungarian population (*N* = 1200, unpublished raw data) investigated with identical tools for pathological distress and sense of coherence. The two samples were not different in terms of age, gender, place of residence, and marital status by chi-square goodness of fit test showing their demographic comparability. 23.25% more rehabilitated individuals experienced pathological distress compared to the population sample (*p* = 0.010) whereas sense of coherence was significantly, 10.53 points lower in our sample than in the population sample (*p* = 0.014) (see Table 2).

**Table 2:** Comparison of the rehabilitated sample with a representative sample of the Hungarian population

	Rehabilitated *N* = 19 (2023)	Representative population sample *N* = 1200 (2023)	*p*
Age (year, mean ± SD)	53.95 (± 12.95)	49.46 (± 1.22)	0.148
Gender (male, %)	37%	45.75%	0.436
Place of residence (city, %)	75%	68.92%	0.654
Marital status (single, %)	14%	19.18%	0.127
Pathologically distressed (%)	42.1%	18.85%	**0.010**
Sense of coherence (mean score ± SD)	56.50 (± 16.9)	67.03 (± 0.9)	**0.014**

## Discussion

The feasibility of storytelling was tested in a community-based rehabilitation project implemented in eight sessions for persons living with mental disorders. The method proved to be feasible based on the voluntary participation rate between 31 and 49% with core group members consistently attending most sessions.

Real-life stories received higher mean scores in terms of being interesting and comprehensible in comparison to fictional (mythical) stories. Statistical analysis revealed two correlations: one indicating that higher scores of the stories being interesting corresponded to increased comprehensibility, and longer stories expressed in word counts were related to decreased comprehensibility. However, based on all sessions, both mean scores characteristic of the stories (being interesting and being comprehensible) were high, above 7 on a 0–10 scale. Participants consistently expressed their desire to join the next session, and by the end, several participants became interested in individual psychotherapy. The investigated mental variables of the participants had remained less favorable compared to the general population but their life satisfaction did increase by the end of the project.

The pre-selected stories served as narratives setting the topic(s) for the session. Stories were told as a series of events occurring to a protagonist to whom participants could relate in some way. All stories involved difficult life events that presented problems for the protagonist; all involved persons in contact with the protagonist who made important decisions typically without informing or involving the protagonist. We used a non-directive approach commonly used with persons with schizophrenia [51] to help them express themselves whatever content was elicited by the story. As it happens, the contribution of some patients during discussions seemed to be quite distant but the authors who conducted the sessions deliberately aimed at connecting even seemingly unrelated points in some way into the group discussion. Recurrent topics emerged in almost all sessions and included intimate partner violence, adverse childhood and adulthood experiences.

Amongst the strengths of our study is its novel approach of using mythical or real-life stories which generated interest and consistently provoked a wide range of thoughts and feelings in rehabilitated patients with mental health issues over four months, leading to improved life satisfaction. Since participation was restricted to members of the Association of Psychotic Patients, the number of participants was limited, therefore the results cannot necessarily be extrapolated to other patients with mental health issues. Another limitation may be that feedback from participants was limited to single-item questions which would have been expanded by structured interviews at the end of the session. However, time and resource constraints did not allow this approach in our project. In spite of its limitations, our study is a meaningful contribution to the scarcity of research articles on this topic.

Storytelling is related to narrative therapy of which Carl Jung [52] can be considered a forefather on account of his work on mythology which he found to be populated by archetypes or primordial patterns of human characters.

“Narrative therapy” was formally developed and operationalized by Michael White and David Epston at the end of the 1980s [53]. They were inspired by a number of theorists, among them Bruner [39], the father of narrative psychology who was the first to discern paradigmatic and narrative thinking, proposing that the latter creates our lived interpretations of reality in the form of stories. Another theorist acknowledged by White and Epson is Foucault who posited a tight relationship between knowledge and power [54] that extends to everyday discourses in which– among others– personal identities and mental problems are created.

James Pennebaker is another researcher to be mentioned who was the first to uncover the therapeutic effect of constructing a written story of traumatic events [55] which served as the basis of his writing therapy used for healing and growth.

White and Epston, working with a wide range of groups, helped participants “re-author” their lives including their trauma along with the participants’ responses. The two authors themselves questioned the appropriateness of the word “therapy” for their work since they did not construct problems in terms of diseases so they also found the concept of “cure” inadequate. Instead, they separate the problem from the person and help narratively recreate the experiences of the person with the problem.

Our approach was similar to theirs in that respect that we did not think of storytelling as “therapeutic” but rather health-promoting that enabled the participants to relate to the protagonists, fictionary or real, of the stories to which they could connect. Our project was different from that of White and Epson since sessions started with stories told by the authors (professionals) instead of stories brought by the participants. We chose this approach because our previous experiences led us to suppose that sharing traumatic life stories even in groups of people knowing each other can result in awkward silence or one or two persons taking the lead. Our approach proved to be appropriate for eliciting life stories of participants in which they covered not only their difficulties but the various ways they dealt with them.

In terms of the stories chosen, their length in word count, complexity including the number of important persons in the story, and the time-span covered should all be taken into account for selecting stories.

Considering additional therapeutic modalities used with persons with schizophrenia in particular, or for those with mental disorders in general, a wide range of psychological interventions have been tested. Arts therapies were found to be effective in reducing negative symptoms and therefore are recommended by NICE guideline [56] but the systematic review of Attard and Larkin concluded that more quantitative evidence would be needed to provide conclusive evidence [25].

Distinct within arts therapies is bibliotherapy or book therapy, usually applied as supportive intervention in which a wide variety of texts tailored to the specifics of the patient groups may be used [57].

A similar, narrative-based but rigorously operationalized program is the metacognitive training of Moritz et al. which focuses on teaching patients to re-learn cognitive errors in two parallel cycles of ten modules for small patient groups in approximately one-hour sessions [58]. Its implementation is aided by well-structured training materials including a host of carefully crafted narrative scenarios by which various types of cognitive errors can be identified and discussed. MCT was shown to reduce symptom severity and improve cognitive functions in patients with schizophrenia [59] an/or psychosis [60] but its application is tested only in clinical patients, requires trained personnel, training materials, and precise planning. The conditions required for the implementation of MCT were not available in our setting.

## Conclusion

We provide evidence for the feasibility of storytelling as a means for health promotion in a group of rehabilitated patients with mental health issues. Significant improvement in life satisfaction was shown in the space of four months in this group though their stress level and sense of coherence remained below the level of their peers in the general population. Carefully selected and narrated stories can elicit a range of thoughts and feelings in participants that may result in the gradual shift of their perspectives and even reconstruction of their life stories that improve life satisfaction. Given the anonymous and voluntary nature of participation or withdrawal, no harmful effects had been predicted and none were experienced.

## Electronic supplementary material

Below is the link to the electronic supplementary material.

Supplementary Material 1

Supplementary Material 2

## Data Availability

The datasets generated and/or analysed during the current study are not publicly available due to ethical restriction but are available from the corresponding author on reasonable request.
