# Supplementary material for: Storytelling as narrative health promotion in community psychiatry: a quasi-experimental study
Source: BMC Psychiatry. 2025 Apr 14;25:376. doi: 10.1186/s12888-025-06816-1 (PMC11995612; doi:10.1186/s12888-025-06816-1)
Supplement: Supplementary file 1 — Supplementary Material 1 [file 12888_2025_6816_MOESM1_ESM.docx]

Appendix 1. Variables and feedback indicators of the sessions by interest and comprehensibility

|  | **Session** | **Word count** | **N** | **Missing** | **Mean** | **SD** | **Min** | **Max** |
| --- | --- | --- | --- | --- | --- | --- | --- | --- |
| **Story found interesting** | 1 | 5301 | 19 | 0 | 8.16 | 2.035 | 4 | 10 |
|  | 2 | 1047 | 12 | 0 | 8.92 | 1.929 | 4 | 10 |
|  | 3 | 1258 | 17 | 0 | 9.24 | 1.091 | 7 | 10 |
|  | 4 | 10602 | 15 | 0 | 8.40 | 2.131 | 3 | 10 |
|  | 5 | 950 | 16 | 0 | 9.44 | 0.892 | 7 | 10 |
|  | 6 | 555 | 14 | 0 | 9.07 | 1.269 | 6 | 10 |
|  | 7 | 716 | 14 | 0 | 9.43 | 0.756 | 8 | 10 |
|  | 8 | 939 | 14 | 1 | 8.93 | 2.018 | 3 | 10 |
| **Story found comprehendible** | 1 | 5301 | 19 | 0 | 7.37 | 2.314 | 2 | 10 |
|  | 2 | 1047 | 12 | 0 | 8.33 | 2.146 | 4 | 10 |
|  | 3 | 1258 | 17 | 0 | 9.06 | 1.519 | 5 | 10 |
|  | 4 | 10602 | 15 | 0 | 8.21 | 2.860 | 1 | 10 |
|  | 5 | 950 | 16 | 0 | 8.88 | 1.204 | 6 | 10 |
|  | 6 | 555 | 14 | 0 | 9.00 | 1.414 | 5 | 10 |
|  | 7 | 716 | 14 | 0 | 9.07 | 1.439 | 6 | 10 |
|  | 8 | 939 | 14 | 1 | 9.71 | 0.611 | 8 | 10 |
