# Supplementary material for: Storytelling as narrative health promotion in community psychiatry: a quasi-experimental study
Source: BMC Psychiatry. 2025 Apr 14;25:376. doi: 10.1186/s12888-025-06816-1 (PMC11995612; doi:10.1186/s12888-025-06816-1)
Supplement: Supplementary file 2 — Supplementary Material 2 [file 12888_2025_6816_MOESM2_ESM.docx]

Appendix 2. Descriptives of the standardized questionnaires

|  |  |  |  |  |  |  |  |  | **Shapiro-Wilk** | |
| --- | --- | --- | --- | --- | --- | --- | --- | --- | --- | --- |
|  |  | **N** | **Missing** | **Mean** | **Median** | **SD** | **Min** | **Max** | **W** | **p** |
| **Before** | GSE-10 | 10 | 1 | 26.7 | 28 | 7.57 | 12 | 38 | 0.895 | 0.195 |
|  | SOC-13 | 11 | 0 | 54.3 | 56 | 19.53 | 19 | 81 | 0.934 | 0.458 |
|  | GHQ-12 | 11 | 0 | 26.7 | 23 | 9.79 | 14 | 45 | 0.941 | 0.528 |
|  | LS | 10 | 1 | 5.40 | 5.50 | 2.50 | 1 | 9 | 0.965 | 0.842 |
| **After** | GSE-10 | 11 | 0 | 26.4 | 29 | 8.23 | 11 | 35 | 0.817 | 0.016 |
|  | SOC-13 | 11 | 0 | 56.6 | 56 | 16.75 | 18 | 82 | 0.913 | 0.262 |
|  | GHQ-12 | 11 | 0 | 26.0 | 24 | 9.44 | 12 | 39 | 0.929 | 0.397 |
|  | LS | 10 | 1 | 6.60 | 6.50 | 2.50 | 1 | 10 | 0.918 | 0.337 |
